# An innovative anticonvulsant - a GABAA receptor modulator with an alternative mechanism of action and enzyme-inducing detoxifying properties

**DOI:** 10.1192/j.eurpsy.2021.2057

**Published:** 2021-08-13

**Authors:** T. Shushpanova, N. Bokhan, K. Stankevich, T. Novozheeva, O. Shushpanova, V. Udut, N. Garganeeva, E. Markova, E. Knyazeva, S. Safronov, R. Boev, A. Solonskii

**Affiliations:** 1 The Department Of Addictive States, Mental Health Research Institute, Tomsk National Research Medical Center of the Russian Academy of Sciences, Tomsk, Russian Federation; 2 Department Of Psychiatry, Narcology And Psychotherapy, Siberian State Medical University, Tomsk, Russian Federation; 3 Institute Of Physics Of High Technologies, National Research Tomsk Polytechnic University, Tomsk, Russian Federation; 4 Neurobiology, Mental Health Research Institute Tomsk National Research Medical Center Russia Academy of Science, Tomsk, Russian Federation; 5 Child Psychiatry, Mental Health Scientific Center, Moscow, Russian Federation; 6 Pathophysiology And Pharmacology,Scientific Research Institute of Pharmacology and Regenerative Medicine named after E. D. Goldberg “Tomsk National Research Medical Center of the Russian Academy of Sciences”, Tomsk, Russian Federation; 7 Department Of Outpatient Therapy, Siberian State Medical University, Tomsk, Russian Federation; 8 Neuroimmunology Lab, State Research Institute of Fundamental and Clinical Immunology, Novosibirsk, Russian Federation

**Keywords:** anticonvulsant, cytochrome, receptor, homeostasis, neuromorphology

## Abstract

**Introduction:**

The development of original drugs - new generation GABAA receptor modulators (GABAAR), with an anti-alcohol orientation, non-addictive and stimulating detoxification processes, makes it possible to increase the effectiveness of therapy and reduce the cost of treatment.

**Objectives:**

Study the mechanism of interaction between m-Cl-BHU and GABAA - receptor

**Methods:**

Molecular docking was performed to study the molecular docking of m-Cl-BHU with at the binding site of the target protein GABAAR.Radioreceptor studies were carried out using [3H] flunitrazepam binding with synaptosomal receptors in the cerebral cortex of Wistar rats in experimental alcoholism under the influence of therapy with m-CL-BHU. Kinetic parameters (T1/2, Clt, MRT, MET, AUC) of a model substrate - antipyrine were determined in the saliva of healthy volunteers and alcoholic patients.

**Results:**

IResults of molecular docking (Schrödinger program (Glide) showed: m-CL-BHU (meta-chlorobenzhydryl urea) is complementary to the benzodiazepine GABAAR. Binding energy is low) (scoring (GScore) -11.14 kKal/mol); m-CL-BHU interacts with key amino acids at the α1γ2 interface: Tyr159, Tyr209, H101 Phe77 and is characterized by a high degree of model fit - dG insert: 0.741 Binding of [3H] flunitrazepam to the benzodiazepine site of GABAAR in rat brain in experimental alcoholism, who received 14 days of m-CL-BHU at 100 mg/kg /day, increased in receptor affinity. Changes in the kinetic parameters (T1/2, Clt, MRT, MET, AUC) of a model substrate - antipyrine in the saliva of healthy volunteers and alcoholic patients using Galodif (m-CL-BHU) at 300 mg/day 21 days

**Conclusions:**

m-CL-BHU - GABAA receptor modulator with an alternative mechanism of action

**Disclosure:**

No significant relationships.

